# 1701. *Trends in Invasive Candidiasis in Children with Hematologic Malignancies: Increased Risk of Dissemination with Non-albicans Candida*

**DOI:** 10.1093/ofid/ofad500.1534

**Published:** 2023-11-27

**Authors:** Amira Said, Faraz Afridi, Chelsea Daignault, Candelaria O’Farrell, Michael E Scheurer, Michele S Redell, Natalie J Dailey Garnes, Maria Gramatges, Ankhi Dutta

**Affiliations:** Baylor College of Medicine, Houston, Texas; MD Anderson Cancer Center, Houston, Texas; Baylor College of Medicine/Texas Children's Hospital, Houston, Texas; Baylor College of Medicine, Houston, Texas; Baylor College of Medicine, Houston, Texas; Baylor College of Medicine, Houston, Texas; University of Texas MD Anderson Cancer Center, Houston, TX; Baylor College of Medicine, Houston, Texas; Baylor College of Medicine, Houston, Texas

## Abstract

**Background:**

Candida species are the most common cause of invasive fungal infections in hospitalized patients. Children with hematological malignancies are at increased risk. The epidemiology of species implicated in invasive candidiasis (IC) has changed over the past 25 years with non-albicans Candida (NAC) species now prevailing. These changes have therapeutic and prophylactic implications as some species carry intrinsic resistance to widely-used antifungal medications.

**Methods:**

Patients ages 0-18 with hematologic malignancy and at least one episode of IC from 2011-2022 from two institutions were included. Patients who had received a bone marrow transplant were excluded. Information related to demographics, host factors, clinical risk factors, *Candida* species and antifungal minimum inhibitory concentrations (MICs), treatment and outcomes was collected via retrospective chart review.

**Results:**

53 cases of IC were identified. Sixty percent were males. Thirty (57%) were of Hispanic ethnicity. The median age at diagnosis was 9 years (range 2 months-18 years). Seventy percent (37/53) had B-cell acute lymphoblastic leukemia. The median time to IC from diagnosis of cancer was 95 days (IQR 20-277). *C. tropicalis* (n=17; 32%) and *C. albicans* (n=14; 26%) were the most common species followed by *C. glabrata* and *C. parapsilosis*. Thirty-five percent (18/53) had concurrent bacteremia. The most common sites of dissemination were the lungs (n=24, 45%), kidneys (n=17; 32%), liver (n=15, 28%) and spleen (n=14; 27%). Of the 35 patients who underwent dilated eye exams, 23% (n=8) had ophthalmologic involvement. NAC were more likely to disseminate to the eyes, skin and spleen than *C. albicans* (p< 0.05). Fourteen isolates (26%) demonstrated increased MICs to fluconazole and six demonstrated increased MICs to voriconazole. Most (59%) required more than 6 weeks of antifungals. Sixteen patients died, of which six were attributed to IC.

**Conclusion:**

NAC accounted for most of the IC in hematological malignancies with *C.tropicalis* being the most common. The implications of NAC being predominant in this high-risk group is concerning due to the higher MIC to azoles and the potential for azole resistance in the future. Antifungal prophylaxis and treatment both need to be reevaluated periodically based on these findings.

**Disclosures:**

**Natalie J Dailey Garnes, MD, MPH**, AlloVir: Grant/Research Support

